# Efficient data selection for time series forecasting using a lightweight linear proxy framework

**DOI:** 10.1038/s41598-026-43645-y

**Published:** 2026-04-09

**Authors:** Xiang Ao, Mengru Chen

**Affiliations:** School of Software Engineering, Beijing Jiaotong University, Beijing, 100044 China

**Keywords:** Time series forecasting, Influence estimation, Robust learning, Transfer learning, Engineering, Mathematics and computing

## Abstract

Time series forecasting is pivotal in domains such as finance, transportation, and meteorology. In practical engineering applications, model performance heavily hinges on the quality and quantity of data. However, the dual challenges of noise and redundancy in large-scale datasets, coupled with data scarcity in specific scenarios, remain significant hurdles. While traditional data valuation methods aim to select high-quality samples, they often require computationally prohibitive gradient calculations, rendering them infeasible for complex deep-time series models. To address these issues, this paper proposes a unified data selection framework based on a Linear Proxy and Mirrored Influence. Motivated by the finding that linear models can efficiently capture core low-frequency temporal trends, we employ a lightweight linear proxy to rapidly evaluate sample value. This approach uses solely lightweight forward passes, thereby circumventing expensive gradient calculations while maintaining selection accuracy. The proposed method achieves two core functions within a unified architecture. Firstly, for standard training scenarios, we design an in-domain pre-selection mechanism guided by a validation set. This mechanism effectively identifies and eliminates detrimental samples prior to training, significantly enhancing both the training efficiency and prediction accuracy of the subsequent main model. Secondly, for few-shot scenarios, we propose a cross-domain data retrieval strategy. Leveraging limited target domain data as guidance, this strategy adaptively selects beneficial samples with consistent distributions from a large-scale source domain pool, effectively mitigating the data scarcity problem. Extensive experiments demonstrate that our method effectively resolves the challenges of training set denoising and cross-domain data augmentation while significantly reducing computational costs.

## Introduction

Time series forecasting stands as a cornerstone of modern data science, underpinning critical decision-making processes in diverse fields. Beyond traditional applications like financial market analysis^1,2^, traffic flow management^3^, and meteorological disaster early warning systems^4^, it is pivotal in energy grid load planning^5^, supply chain demand estimation^6^, anomaly detection in AIOps^7^, climate change analytics^8^, and industrial predictive maintenance^9^. With the advent of deep learning, the field has witnessed a paradigm shift from traditional statistical methods (e.g. ARIMA^10^) to complex neural architectures like Transformers^11^ and their variants (e.g. Autoformer^12^). While these advanced models possess potent feature extraction capabilities, their predictive efficacy is intrinsically tied to the scale and quality of the underlying training data^13^. Recently, the focus of the community has gradually expanded from Model-Centric AI, which pursues complex architectures to Data Centric-AI^14^, emphasizing the refinement of data quality. However, the transition from academic research to real world engineering deployment is fraught with challenges, primarily stemming from the inherent imperfections and data cascades often found in industrial environments^15^.

These challenges manifest in two distinct yet interconnected aspects: *data redundancy* and *data scarcity*. First, in data-rich scenarios, large-scale datasets are frequently contaminated with noise, outliers, and redundant samples^16^. Unlike image or text data, time series data possesses continuous temporal dependencies, making it difficult to distinguish between meaningful “hard samples” and detrimental noise using simple heuristics (e.g. loss thresholding). Such low-quality data not only imposes a significant computational burden during training but also predisposes complex models to suboptimal convergence and the phenomenon of “negative transfer”^17^. Second, in emerging applications or cold-start scenarios, high-quality target domain data is often critically scarce (Data Scarcity)^18^. Deep forecasting models typically require vast amounts of data to learn robust temporal representations. When data is insufficient, they are prone to overfitting. While transfer learning offers a potential solution, blindly migrating models trained on heterogeneous source domains can lead to performance degradation if the source and target dynamics are misaligned.

Despite the urgent need for high-quality data, effective data selection in time series remains an open problem. While theoretical frameworks for data valuation–such as gradient-based Influence Functions^19^, TracIn^20^, and Data Shapley^21^–offer a means to quantify sample contributions, their practical utility is severely limited by computational prohibitiveness. Specifically, these methods typically require expensive Hessian matrix inversions or extensive model retraining. For long-sequence time series models with millions of parameters, this incurs an $O(NM^2)$ complexity (where *M* is the parameter size) that is untenable in industrial settings requiring real-time scalability. Conversely, lightweight heuristic methods (e.g. selecting samples with small losses) often lack theoretical guarantees and fail to capture the global distribution characteristics required for generalization.

To bridge this gap between theoretical data valuation and practical efficiency, this paper proposes LP-Mirror, a unified data selection framework grounded in the Linear Proxy architecture and the Mirrored Influence hypothesis(MIH)^22^. The core motivation is to decouple the expensive influence estimation process from deep model training. Inspired by recent findings that simple linear models can effectively capture core temporal trends^23,24^, we introduce a lightweight linear proxy to substitute complex deep backbones during the selection phase. Crucially, to utilize the inherent temporal characteristics of time series data, this linear proxy acts as a robust filter by fitting the dominant “low-frequency” temporal components, such as trends and seasonality. By evaluating whether a sample’s temporal gradient aligns with the guide set, it can effectively identify and distinguish meaningful temporal dynamics from distorted trends or high-frequency noise . This proxy captures essential temporal dynamics solely through efficient forward passes, thereby circumventing the burdensome backward gradient calculations associated with traditional methods. Our approach is predicated on the MIH, utilizing a validation or target set as a benchmark to infer the marginal utility of training samples by observing loss perturbations in the proxy model. Within this unified architecture, we specifically operationalize data selection across two distinct scenarios based on these temporal evaluations. Firstly, for standard training regimes, we employ an in-domain pre-selection mechanism. By using a clean validation set as the temporal benchmark, this mechanism filters the raw training dataset prior to the primary training phase, effectively exorcising detrimental samples with abnormal temporal fluctuations. Secondly, for few-shot scenarios, we devise a cross-domain data retrieval strategy. Here, the limited target domain data acts as a directional compass, guiding the adaptive retrieval of source samples that share consistent temporal dynamics and distributions, thereby mitigating data scarcity while preventing negative transfer.

Within this unified architecture, we address the aforementioned dual challenges through two specific mechanisms. For standard training regimes, we introduce an *in-domain pre-selection mechanism*. Prior to the primary training phase, this mechanism leverages validation guidance to filter the raw dataset, effectively identifying and exorcising detrimental samples. This ensures that the subsequent deep model concentrates computational resources exclusively on high-value data, achieving a synergy of accelerated convergence and enhanced prediction accuracy. Conversely, for few-shot scenarios, we devise a *cross-domain data retrieval strategy*. Acting as a “compass”, the limited target data guides the adaptive retrieval of distributionally consistent samples from a large-scale source pool, thereby resolving the cold-start problem while mitigating distribution shifts.

The primary contributions of this work are summarized as follows:

- *Unified efficient framework*: We propose a novel data selection framework that synergizes the MIH with a lightweight Linear Proxy. By substituting Hessian-based gradient calculations with rapid forward passes, this method dramatically reduces computational overhead, enabling scalable data valuation for long-sequence time series tasks.
- *Validation-guided denoising*: We design a robust pre-selection strategy that identifies and eliminates harmful or redundant samples prior to model training. This mechanism ensures focus on high-value data subsets, significantly improving prediction accuracy while simultaneously accelerating training convergence.
- *Cross-domain retrieval*: To tackle data scarcity, we develop an adaptive cross-domain retrieval strategy. By leveraging minimal target data to navigate large-source pools, this approach effectively solves the cold-start problem and prevents negative transfer by filtering for dynamic consistency.
- *Empirical validation*: Extensive experiments across four mainstream model architectures validate the versatility and effectiveness of our approach. The results demonstrate improved performance in both full-data training and few-shot transfer tasks, confirming the framework’s potential as a general-purpose solution for time series data optimization.

## Related work

### Time series forecasting: from complex architectures to high-efficiency paradigms

With the rapid advancement of deep learning, the architecture of time series forecasting models has undergone a paradigm shift from Recurrent Neural Networks (RNN)^25^ to Transformers, and recently, towards diverse efficient architectures. Early research primarily relied on LSTMs^26^ or GRUs^27^ to capture temporal dependencies, yet these recurrent structures often suffer from gradient vanishing problems in long-sequence modeling.

In recent years, Transformer architectures have achieved dominance due to their global receptive fields. Autoformer^12^ introduced a decomposition architecture with an auto-correlation mechanism to handle long-range dependencies efficiently. Fedformer^28^ further reduced computational complexity to linear scale by incorporating frequency domain analysis and mixture-of-experts blocks. Building on the patching technique, PatchTST^29^ proposed channel independence and patch-based attention, significantly improving prediction accuracy by preserving local semantic information. To address the issue of ignoring cross-variate correlations in channel-independent(CI) models, Crossformer^30^ and iTransformer^31^ were proposed in 2023 and 2024 respectively. Specifically, iTransformer inverts the dimensions of the Transformer, embedding the entire time series of each variate as a token, thereby effectively capturing multivariate correlations while maintaining efficient computation.

Parallel to the development of dedicated architectures, the adaptation of Large Language Models (LLMs)^32^ for time series has emerged as a burgeoning trend. Methods like Time-LLM^33^ and Lag-Llama^34^ align time series embeddings with the semantic space of pre-trained LLMs, demonstrating strong zero-shot generalization capabilities. However, the immense parameter size and inference latency of these foundation models pose significant hurdles for resource-constrained industrial deployment.

Conversely, a wave of research has begun to revisit the effectiveness of simple architectures, challenging the necessity of complex attention mechanisms. TimesNet^35^ transforms 1D time series into 2D tensors to capture multi-periodicity using standard convolutions. Zeng et al. proposed DLinear^23^, demonstrating that after trend and seasonal decomposition, simple linear models can outperform complex Transformers on multiple benchmarks. This “simplicity” trend has been further expanded: ModernTCN^36^ revitalized convolutional structures with large kernel designs to achieve excellent performance with lower overhead, while FITS^37^ utilizes complex-valued linear interpolation in the frequency domain to achieve parameter-efficient forecasting. TimeMixer^24^ and the recent WPMixer^38^ perform multiscale mixing of past information to enhance MLP capabilities. Similarly, TimeMachine^39^ leverages State Space Models (Mamba^40^) to achieve linear complexity. These findings provide theoretical support for our approach–utilizing lightweight linear proxies for rapid data assessment is not merely a compromise, but a strategy grounded in the proven efficacy of simplified structures.

### Data selection and influence estimation

Data quality is paramount for model generalization, especially in time series where distribution shifts are prevalent. The Influence Functions proposed by Koh and Liang^19^ serve as a foundational method, approximating the effect of Leave-One-Out (LOO) retraining by calculating the inverse Hessian matrix. However, the $O(NM^2)$ complexity renders it infeasible for modern deep models.

To circumvent expensive overhead, research has explored various approximation paths. One direction involves gradient-based valuations such as TracIn^20^ and Data Shapley^21^, which trace gradient descent trajectories. Another direction focuses on lightweight heuristics, such as loss-based selection and dynamic pruning algorithms like Moderate-DS^41^ and InfoBatch^42^. In the context of large-scale fine-tuning, LESS^43^ recently proposed selecting influential data by projecting gradients into a low-dimensional validation space, significantly reducing computational costs.

Despite these advancements, most selection methods assume i.i.d. data and lack specific designs for temporal dependencies. In practical industrial applications, the complexity of time series data–such as dynamic distribution shifts, spatial correlations, and imbalanced data distributions–has increasingly become a bottleneck, prompting various specialized data-driven mitigation strategies (e.g. resampling and dynamic modeling in fault diagnosis)^44–46^. Addressing the non-stationary nature of time series in general forecasting tasks, Dish-TS^47^ highlights the impact of distribution shift on forecasting, suggesting that data selection must account for distributional consistency. Furthermore, SoftPatch^48^ introduces a noise-rejection mechanism specifically for patch-based time series models.

To balance theoretical rigor with efficiency, Ko et al.^22^ proposed the MIH, demonstrating that the influence of training data on test predictions is symmetric to the influence of test data on the training model. This allows replacing burdensome gradient calculations with efficient Forward Passes. While effective in static domains, exploring how to adapt this mirror mechanism to the continuous and redundant nature of time series data remains an open challenge. This paper addresses this gap by proposing the LP-Mirror framework.

## Methodology

**Fig. 1:**
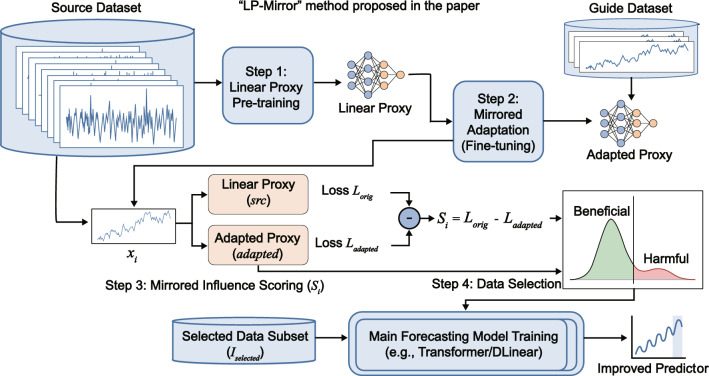
Overview of the LP-mirror framework. The pipeline consists of four sequential steps: (1) Linear Proxy Pre-training on the source dataset, (2) Mirrored Adaptation on a small guide dataset, (3) Mirrored Influence Scoring to quantify gradient alignment, and (4) Rank-based Data Selection. The specific definition of the Guide Dataset flexibly adapts to different application scenarios.

In this section, we propose the LP-Mirror framework, a novel “data-centric” paradigm designed to enhance time series forecasting capabilities through the rigorous filtration of training data. As illustrated in Fig. 1, the overall pipeline unfolds through four interconnected phases. It begins with pre-training a linear proxy on the source dataset to capture foundational temporal dynamics. Following this, a mirrored adaptation process performs few-shot fine-tuning on a small guide dataset to internalize the target distribution. By calculating the loss difference between the pre-trained and adapted proxies, we establish a mirrored influence score for each source sample that directly quantifies its gradient alignment. Ultimately, a rank-based selection module filters the samples according to these derived scores.

We first formulate the data selection problem as a bi-level optimization task. Next, we detail the Channel-Independent (CI) Linear Proxy architecture, designed to capture temporal dynamics efficiently. Subsequently, we derive the MIH-based influence estimation mechanism, providing a theoretical connection to gradient alignment. Finally, we analyze the computational complexity and present the implementation algorithm.

### Problem formulation

#### Multivariate time series forecasting

Let $\mathscr {D} = \{(\textbf{x}_i, \textbf{y}_i)\}_{i=1}^N$ denote a large-scale historical dataset, which may be contaminated by noise, non-stationary shifts, or outliers. Here, $\textbf{x}_i \in \mathbb {R}^{L \times C}$ represents a look-back window of length *L* across *C* variables, and $\textbf{y}_i \in \mathbb {R}^{P \times C}$ represents the ground truth prediction horizon of length *P*. We assume the existence of a small, high-quality guide set $\mathscr {D}_{guide}$ (e.g. a validation set or limited samples from the target domain) that represents the ideal data distribution $\mathscr {P}_{target}$.

#### A bi-level optimization perspective

The goal of data selection is to determine a weight vector $\textbf{w} = (w_1, w_2,\dots , w_N)$ that selects a subset of $\mathscr {D}_{train}$ to maximize the model’s performance on $\mathscr {D}_{guide}$. This can be rigorously formulated as a bi-level optimization problem, where the outer loop minimizes the validation loss and the inner loop minimizes the weighted training loss:

1 $$\begin{aligned} \begin{aligned}&\min _{\textbf{w}} \quad L_{val}(\phi ^*(\textbf{w}); \mathscr {D}_{guide}) := \frac{1}{|\mathscr {D}_{guide}|} \sum _{(\textbf{x}_v, \textbf{y}_v) \in \mathscr {D}_{guide}} \ell (f_{\phi ^*(\textbf{w})}(\textbf{x}_v), \textbf{y}_v) \\&\text {s.t.} \quad \phi ^*(\textbf{w}) = \underset{\phi }{\arg \min }\ \left( \sum _{i=1}^N w_i \cdot \ell (f_\phi (\textbf{x}_i), \textbf{y}_i) + \lambda \mathscr {R}(\phi ) \right) \\&\quad \quad \ \ \textbf{w} \in \{0, 1\}^N \end{aligned} \end{aligned}$$

here, $f_\phi$ is the forecasting model (e.g. Transformer, LSTM), $\ell (\cdot )$ denotes the sample-wise loss function, and $\lambda \mathscr {R}(\phi )$ represents the regularization term. The notation $\phi ^*(\textbf{w})$ indicates the optimal parameter solution obtained by minimizing the weighted training loss for a fixed $\textbf{w}$. Directly solving this problem is computationally infeasible, as it requires retraining $f_\phi$ (i.e. recomputing $\phi ^*$) for every change in $\textbf{w}$. LP-Mirror circumvents this issue by approximating the sensitivity of the inner optimization loop using a lightweight proxy.

### The linear proxy

Standard deep models (e.g. PatchTST, iTransformer) typically involve complex attention mechanisms ($O(L^2)$) and significant GPU memory consumption, making them unsuitable for iterative influence estimation. To ensure scalability, we design a Channel-Independent (CI) Linear Proxy.

In our implementation, the proxy treats multivariate series as independent univariate channels. The input tensor $\textbf{X} \in \mathbb {R}^{B \times L \times C}$ is permuted to $\textbf{X}' \in \mathbb {R}^{B \times C \times L}$. This aligns the time dimension *L* as the feature dimension for the linear layer. A single linear layer with weights $\theta \in \mathbb {R}^{L \times P}$ and bias $\textbf{b} \in \mathbb {R}^P$ is applied to the last dimension. Crucially, weights are shared across all *C* channels to prevent overfitting and enforce consistent temporal modeling:

2 $$\begin{aligned} \hat{\textbf{Y}}' = \textbf{X}' \theta + \textbf{b} \quad \in \mathbb {R}^{B \times C \times P} \end{aligned}$$

besides, we denote the parameters of the linear proxy model as $\theta _{p} = \{\theta ,b\}$. Let $\theta _{src}$ and $\theta _{adapted}$ denote two snapshots of $\theta _{p}$ before and after mirror adaptation, respectively. Finally, the output is permuted back to $\hat{\textbf{Y}} \in \mathbb {R}^{B \times P \times C}$ to match the ground truth format.

#### Why is the linear proxy effective?

Despite its simplicity, the linear model effectively captures the “low-frequency” components (trend and seasonality) of time series data. According to the spectral bias theory of neural networks, noise typically manifests as high-frequency irregularities^49^. If a sample’s trend is heavily distorted (outliers) or reversed (distribution shift), a linear model fitting the dominant trend will exhibit high residuals or produce gradients that conflict with the clean guide set. Thus, the linear proxy acts as a robust “high-pass filter” for detecting data quality issues.

*Discussion on highly nonlinear sequences*: We acknowledge that for highly nonlinear time series, the linear proxy may inevitably underfit complex high-frequency dynamics. However, in the context of data selection, this underfitting acts as a conservative safety mechanism. By strictly filtering based on the alignment of dominant low-frequency macro-trends, the linear proxy ensures that samples with contradictory foundational dynamics (severe distribution shifts) are discarded. While it might conservatively filter out some informative high-frequency nonlinear patterns, it effectively guarantees a high-purity subset, providing a robust and safe initialization for subsequent high-capacity nonlinear backbones (e.g. Transformers) to learn complex patterns without being misled by foundational noise. This is empirically validated by our significant performance gains on highly complex and nonlinear datasets such as Traffic and Electricity.

*Robustness to guide set quality and size*: A critical advantage of the linear proxy is its intrinsic resilience against potential biases or limited sizes of the guide set. Instead of overfitting to the idiosyncratic noise or skewness present in a small guide set, the linear proxy fundamentally establishes a “baseline standard” representing the global macroscopic trends. Because linear models possess low sample complexity, capturing this foundational temporal relationship requires only a minimal amount of data (e.g. merely 10% of the target domain). Consequently, even if the guide set is extremely small or contains minor distributional biases, the linear proxy resists propagating these flaws. It ensures that the gradient alignment process evaluates structural temporal consistency rather than overfitting to specific localized noise, thereby avoiding bias propagation.

### MIH-based influence estimation

To efficiently quantify the value of training samples without the prohibitive cost of retraining or computing inverse Hessians, we leverage the *Mirrored Influence Hypothesis (MIH)*^22^. Traditional influence functions ask: “How would the test loss change if we upweighted this training sample?” This typically requires second-order optimization information. MIH simplifies this by assuming a symmetry: the influence of a training sample on the model’s test performance is approximately equal to the influence of a (test/guide set-driven) model update on that training sample’s loss.

#### Derivation

Let $\theta _{src}$ be the parameters of the proxy model pre-trained on the source dataset $\mathscr {D}_{train}$. Rather than directly calculating the impact of training data on validation loss (which is expensive), we simulate a “look-ahead” step. We perform a virtual, one-step update using the guide set $\mathscr {D}_{guide}$ with a small learning rate $\eta _{p}$:

3 $$\begin{aligned} \theta _{adapted} = \theta _{src} - \eta _{p} \nabla _\theta L(\mathscr {D}_{guide}; \theta _{src}) \end{aligned}$$

where $\nabla _\theta$ denotes the gradient operator with respect to $\theta$, and $L(\cdot )$ represents the aggregated loss function calculated on $\mathscr {D}_{guide}$ (e.g. Mean MSE).

Next, we denote the *i*-th sample in the source dataset as $z_i = (\textbf{x}_i, \textbf{y}_i)$. We define the *Influence Score* $S_i$ for this sample as the reduction in its individual loss after the model has undergone the target-driven update described above. Intuitively, if the model predicts $z_i$ better after learning from the guide set (i.e. loss decreases, so $S_i > 0$), it implies that $z_i$ shares useful knowledge with $\mathscr {D}_{guide}$:

4 $$\begin{aligned} S_i = \ell (z_i; \theta _{src}) - \ell (z_i; \theta _{adapted}) \end{aligned}$$

To reveal the theoretical mechanism, we use a first-order Taylor expansion at $\theta _{src}$ to approximate the loss under the new parameters. This approximation holds under the assumption that $\eta _{p}$ is sufficiently small such that the loss surface is locally linear:

5 $$\begin{aligned} \ell (z_i; \theta _{adapted}) \approx \ell (z_i; \theta _{src}) + \nabla _\theta \ell (z_i; \theta _{src})^\top (\theta _{adapted} - \theta _{src}) \end{aligned}$$

Substituting the update rule $\theta _{adapted} - \theta _{src} = -\eta _{p} \nabla _\theta L(\mathscr {D}_{guide})$ into the expansion:

6 $$\begin{aligned} \ell (z_i; \theta _{adapted}) \approx \ell (z_i; \theta _{src}) - \eta _{p} \nabla _\theta \ell (z_i; \theta _{src})^\top \nabla _\theta L(\mathscr {D}_{guide}) \end{aligned}$$

Substituting this approximation back into Eq. (4), the constant terms cancel out, yielding the underlying geometric interpretation:

7 $$\begin{aligned} S_i \approx \eta _{p} \cdot \underbrace{\langle \nabla _\theta \ell (z_i), \nabla _\theta L(\mathscr {D}_{guide}) \rangle }_{\text {Gradient Alignment}} \end{aligned}$$

#### Interpretation and implementation

Equation (7) reveals that the influence score $S_i$ is mathematically equivalent to the dot product between the gradient of the training sample and the average gradient of the guide set.

Geometrically, this dot product measures the directional alignment of the optimization vectors. The value of $S_i$ indicates whether the training sample facilitates or hinders the optimization of the guide set:

- $S_i \gg 0$ (Positive Alignment): The gradient of $z_i$ forms an acute angle with the guide gradient. Training on $z_i$ pushes model parameters in a direction that also minimizes the loss on $\mathscr {D}_{guide}$. These are high-value samples.
- $S_i \ll 0$ (Negative Alignment): The gradients form an obtuse angle or are opposing. Minimizing the loss on $z_i$ increases the loss on the guide set, indicating a conflict (e.g. mislabeled data, outliers, or negative transfer).
- $S_i \approx 0$ (Orthogonal): The sample is irrelevant to the target task.

From a computational standpoint, while Eq. (7) provides theoretical intuition, explicitly computing and storing gradients for every training sample consumes significant memory. Therefore, we employ Eq. (4) for actual calculation. This “forward difference” approach requires only two forward passes per batch (one with $\theta _{src}$, one with $\theta _{adapted}$), thereby completely avoiding explicit gradient instantiation or Hessian computation.

#### Theoretical justification

While the Mirrored Influence Hypothesis (MIH)^22^ was initially explored in computer vision and natural language processing, its validity in continuous time series data requires rigorous theoretical justification. The foundational premise of MIH relies on the mathematical symmetry of the influence function.

Let $H_{\theta _{src}} = \frac{1}{N} \sum _{j=1}^{N} \nabla _{\theta }^2 l(z_j; \theta _{src})$ denote the Hessian matrix of the empirical risk on the source dataset. According to the classic Influence Function theory, the effect of upweighting a training sample $z_i$ on the guide set loss (Train-to-Test Influence) is formulated as:

8 $$\begin{aligned} \mathscr {I}_{train \rightarrow guide}(z_i, \Omega _{guide}) \approx -\frac{1}{N} \nabla _{\theta } L(\Omega _{guide}; \theta _{src})^{\top } H_{\theta _{src}}^{-1} \nabla _{\theta } l(z_i; \theta _{src}) \end{aligned}$$

Conversely, the reciprocal effect of upweighting the guide set $\Omega _{guide}$ on the training sample $z_i$’s loss (Test-to-Train Influence, which our MIH mechanism approximates) is given by:

9 $$\begin{aligned} \mathscr {I}_{guide \rightarrow train}(\Omega _{guide}, z_i) \approx -\frac{1}{N} \nabla _{\theta } l(z_i; \theta _{src})^{\top } H_{\theta _{src}}^{-1} \nabla _{\theta } L(\Omega _{guide}; \theta _{src}) \end{aligned}$$

Because the Hessian matrix $H_{\theta _{src}}$ is symmetric by definition, its inverse $H_{\theta _{src}}^{-1}$ is inherently symmetric. This fundamental linear algebra property guarantees that Eqs. (8) and (9) are mathematically equivalent ($\mathscr {I}_{train \rightarrow guide} \equiv \mathscr {I}_{guide \rightarrow train}$).

Crucially, the alignment with time series modeling lies in the optimization landscape. In practice, this exact mathematical symmetry often deteriorates in highly non-convex deep time series models (e.g. deep Transformers) due to indefinite Hessian matrices and severe local gradient noise. This provides the theoretical imperative for our Linear Proxy design. By utilizing a single linear layer optimized with Mean Squared Error (MSE) loss, the optimization landscape within our framework becomes strictly convex. For a strictly convex formulation, the Hessian matrix is globally positive definite, ensuring that the local linear approximation flawlessly characterizes the loss surface without being perturbed by high-frequency temporal noise. Consequently, the mathematical symmetry of MIH holds exceptionally well for time series data under our LP-Mirror architecture, providing a solid theoretical guarantee for using efficient forward passes to evaluate sample utility.

### Rank-based selection strategy

Based on the calculated scores $\mathscr {S} = \{S_1, \dots , S_N\}$, we adopt a ranking mechanism to determine the final subset. This approach avoids the instability associated with hard thresholding across different datasets.

Specifically, we sort the samples in descending order of their influence scores $S_i$. Let $\text {rank}(i)$ denote the rank of sample *i*. Given a target retention ratio $\rho \in (0, 1]$, the selected index set is defined as

10 $$\begin{aligned} I_{select} = \{i \mid \text {rank}(i) \le \lfloor \rho \cdot N \rfloor \} \end{aligned}$$

Alternatively, in scenarios where data purity is paramount (e.g. high-noise industrial data), we can adopt a strategy that focuses solely on positive influence. We select the subset $\{i \mid S_i > \epsilon \}$, where $\epsilon$ is a small margin, rather than a fixed ratio. This criterion ensures that only samples strictly beneficial to the target task are retained, maximizing precision potentially at the cost of discarding more data.

### Complexity analysis

We compare the computational complexity of LP-Mirror with standard Influence Functions (IF) and TracIn. Let *N* be the dataset size, *M* be the number of parameters, and *H* be the cost of the Hessian-vector product.

**Table 1:** Complexity comparison of data selection methods.

Method	Time complexity	Space complexity	Feasibility
Influence Function	$O(N \cdot H + N \cdot M)$	$O(M^2)$ (Hessian)	Low
TracIn	$O(N \cdot M \cdot K_{cp})$	*O*(*M*)	Medium
**LP-Mirror (Ours)**	$\mathbf {O(N \cdot L \cdot P)}$	$\mathbf {O(1)}$	**High**

As shown in Table 1, standard IF requires computing the inverse Hessian, which is infeasible for Transformer models ($M \approx 10^7$). TracIn necessitates saving multiple checkpoints ($K_{cp}$). In contrast, LP-Mirror utilizes a linear proxy with negligible parameters ($L \times P \approx 336 \times 96 \approx 3 \times 10^4$). The selection process requires only two lightweight forward passes over the dataset, achieving linear time complexity *O*(*N*) with minimal memory overhead, making it easily deployable on standard GPUs.

### Algorithm description

The core selection process is detailed in Algorithm . This module operates efficiently using the lightweight proxy model, allowing for scoring and ranking of samples without substantial computational cost.

**Algorithm 1:**
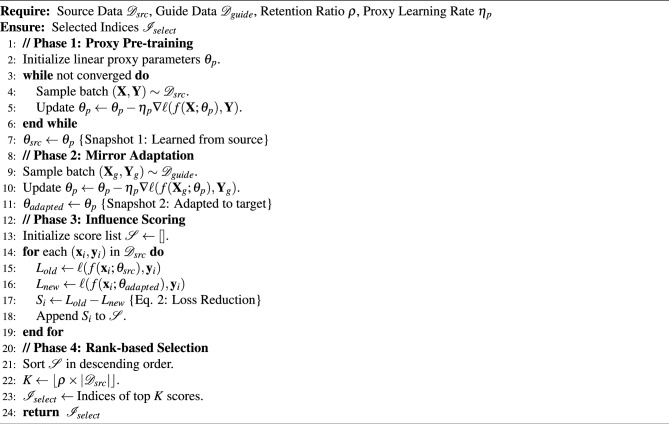
LP-Mirror: core data selection.

## Experiments

To rigorously and comprehensively evaluate the effectiveness, robustness, and computational efficiency of the LP-Mirror framework, we conducted systematic experiments on seven widely used real-world time series datasets. The experimental design centers on addressing core data challenges in time series forecasting, verifying the framework’s performance across multi-dimensional scenarios. Our empirical evaluation aims to explicitly answer the following three Research Questions (RQs):

*RQ1 (Effectiveness)*: Can LP-Mirror consistently improve forecasting accuracy and accelerate the training convergence of downstream backbone models across varying architectures (from lightweight linear models to complex Transformer variants) and two core application scenarios (in-domain denoising and cross-domain few-shot transfer)?

*RQ2 (Superiority)*: Does LP-Mirror demonstrate significant advantages in computational overhead compared to mainstream data selection and influence estimation methods (e.g. gradient-based Influence Functions, TracIn), and does this efficiency extend to large-scale long-sequence datasets?

*RQ3 (Mechanism)*: How does the screening mechanism of LP-Mirror effectively distinguish between beneficial and harmful/redundant samples? We answer this by analyzing influence score distributions, testing hyperparameter sensitivity, and conducting qualitative waveform visualizations to verify the underlying logic.

### Experimental setup

#### Datasets and preprocessing

We selected seven mainstream time series forecasting benchmark datasets covering diverse domains, scales, frequencies, and variable dimensions to fully validate the generalization capability of LP-Mirror. These datasets are derived from real-world scenarios and contain authentic characteristics such as natural fluctuations, noise interference, and outliers. Detailed statistics are presented in Table 2.

**Table 2:** Detailed statistics of the datasets used in experiments.

Dataset	Domain	Variates	Prediction lengths	Total samples	Size (train/val/test)	Frequency
ETTm1	Energy	7	{96, 192, 336, 720}	57507	(34465, 11521, 11521)	15 min
ETTm2	Energy	7	{96, 192, 336, 720}	57507	(34465, 11521, 11521)	15 min
ETTh1	Energy	7	{96, 192, 336, 720}	14307	(8545, 2881, 2881)	Hourly
ETTh2	Energy	7	{96, 192, 336, 720}	14307	(8545, 2881, 2881)	Hourly
Electricity	Power Grid	321	{96, 192, 336, 720}	26211	(18317, 2633, 5261)	Hourly
Weather	Meteorology	21	{96, 192, 336, 720}	52603	(36792, 5271, 10540)	10 min
Traffic	Transportation	862	{96, 192, 336, 720}	17544	(12280, 1756, 3508)	Hourly

Each dataset exhibits distinct domain characteristics. The Energy datasets (ETTm1/ETTm2/ETTh1/ETTh2) include 7 key variables such as electricity load and temperature, sampled at 15-minute or hourly intervals, showing clear periodicity and trends. The Electricity dataset covers hourly power consumption for 321 nodes, characterized by high dimensionality and complex cross-node dependencies with stochastic fluctuations. The Traffic dataset contains traffic flow data for 862 roads; it is large-scale, highly noisy, and contains abnormal patterns like sudden congestion. The Weather dataset records 21 meteorological indicators at 10-minute intervals, featuring uneven distributions and outliers caused by extreme weather. All datasets were preprocessed using mean-variance normalization (Z-score) to eliminate dimensional discrepancies. The input sequence length was unified to 96, with prediction lengths covering short, medium, and long-term scenarios (96/192/336/720 steps).

#### Model architectures and configuration

To verify the framework’s universality, we selected four mainstream time series forecasting models as backbones.

1. *DLinear* a lightweight linear model utilizing trend decomposition and CI forecasting.
2. *PatchTST* a Transformer model based on patch-wise self-attention.
3. *TimeMixer* a multi-scale mixing MLP model.
4. *WPMixer* a multi-resolution wavelet mixing model.

The core parameters for LP-Mirror are set as follows: the linear proxy model uses a CI linear mapping with weight matrix dimension $L \times P$ ($L=96$, *P* is prediction length), and bias initialized to 0. The adaptation learning rate is set to 0.001, and adaptation steps $K=1$ to balance fine-tuning efficiency with adaptability.

All experiments were conducted on a single NVIDIA RTX 4090 GPU (24GB VRAM). The software environment includes PyTorch 2.1.0, CUDA 12.2, and Python 3.9.16. Models were trained using the Adam optimizer with a weight decay of $1\textrm{e}{-4}$. The maximum training epochs were set to 100, utilizing an early stopping strategy (patience=10) to prevent overfitting.

### Performance evaluation (RQ1)

#### In-domain denoising performance

We first verified the accuracy improvements brought by LP-Mirror when cleaning training sets in standard forecasting tasks. Table 3 reports the detailed performance of four backbone models (DLinear, PatchTST, TimeMixer, and WPMixer) on datasets such as ETTh1, ETTm1, and Weather. The results demonstrate that LP-Mirror consistently reduced MSE and MAE across the vast majority of datasets and prediction horizons, exhibiting significant denoising effects.

Regardless of the backbone architecture–whether simple linear models or complex Transformers–LP-Mirror provided performance gains. For instance, on the Weather dataset, the average MSE of the TimeMixer model dropped significantly from 0.222 to 0.212. For DLinear, known for its robustness, LP-Mirror further optimized the average MSE on ETTh1 from 0.423 to 0.415. This indicates that our method is not specific to a single model class but functions as a general-purpose data augmentation plugin.

In challenging long-sequence forecasting scenarios (e.g. prediction length = 720), noisy data often causes error accumulation as the horizon increases. Experimental data shows that for the 720-step prediction on ETTm2, incorporating LP-Mirror reduced WPMixer’s MSE from 0.344 to 0.322, effectively suppressing error divergence in long-term forecasting.

For large-scale datasets with complex noise, such as Traffic and Electricity, LP-Mirror enabled models to focus on high-value subsets by removing abnormal samples. Notably, on the Traffic dataset, it achieved performance improvements of over 10% across all models.

**Table 3:**
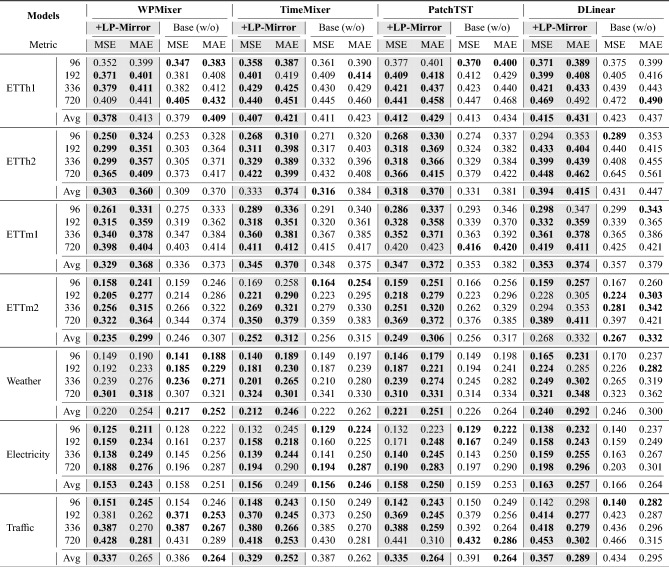
Multivariate long-term forecasting results: Comparison between standard models and models enhanced with LP-Mirror. Shaded columns indicate our method (LP-Mirror). Best results in each pair are bolded.

#### Cross-domain transfer performance

In the cross-domain few-shot scenario, we simulated a “cold-start” problem where target domain data is extremely scarce. Here, a complete Source Domain dataset serves as a candidate pool, while only a small portion of the Target Domain data (retaining only 10%) is used as a guidance set to retrieve beneficial samples. We compared LP-Mirror against two baselines: “Direct Transfer” and “Train on 10% Data (Target Only).” To ensure a strictly fair comparison, we explicitly guarantee that our LP-Mirror method is restricted to accessing exactly the same 10% subset of the target data to serve as its guide set. Our framework has absolutely no access to the remaining 90% of the target data during the entire selection and training pipeline, ensuring that the total information access is strictly identical between our method and the “Train on 10% Data” baseline.

**Table 4:** Transfer learning results on different ETT datasets.

Methods		WPMixer		TimeMixer		PatchTST		DLinear	
		MSE	MAE	MSE	MAE	MSE	MAE	MSE	MAE
ETTh1$\rightarrow$ETTh2	Direct Transfer	0.421	0.418	0.427	0.424	0.380	0.405	0.493	0.488
	Train on 10% Data	0.482	0.481	0.419	0.425	0.449	0.468	0.513	0.514
	Ours	**0.411**	**0.402**	**0.395**	**0.408**	**0.365**	**0.397**	**0.488**	**0.462**
ETTm1$\rightarrow$ETTh2	Direct Transfer	**0.449**	0.437	0.452	0.441	0.439	0.438	**0.464**	**0.475**
	Train on 10% Data	0.482	0.481	0.419	0.425	0.449	0.468	0.513	0.514
	Ours	0.452	**0.432**	**0.412**	**0.412**	**0.377**	**0.402**	0.501	0.489
ETTm1$\rightarrow$ETTm2	Direct Transfer	**0.334**	**0.363**	0.329	0.357	0.296	0.334	0.335	0.389
	Train on 10% Data	0.369	0.425	0.318	0.335	0.334	0.382	0.348	0.395
	Ours	0.338	0.369	**0.306**	**0.312**	**0.267**	**0.311**	**0.298**	**0.337**
ETTh1$\rightarrow$ETTm2	Direct Transfer	0.352	0.391	0.361	0.397	**0.314**	**0.360**	0.415	0.452
	Train on 10% Data	0.355	0.401	0.342	0.398	0.332	0.412	0.348	0.395
	Ours	**0.348**	**0.379**	**0.313**	**0.338**	**0.314**	0.389	**0.301**	**0.345**

The best results for each model are highlighted based on MSE and MAE metrics. The results are the average of 4 prediction lengths.

As shown in Table 4, LP-Mirror achieves optimal performance in most transfer tasks. First, compared to the “Target Only” baseline, LP-Mirror alleviates overfitting caused by data sparsity. For example, in the $ETTm1 \rightarrow ETTm2$ task, the PatchTST model trained on only 10% of the data had a high MSE of 0.334, while data augmentation with LP-Mirror significantly reduced the error to 0.267. This proves our method effectively retrieves high-quality samples from the source domain that are consistent with the target distribution, compensating for the lack of target samples.

Second, compared to “Direct Transfer,” LP-Mirror demonstrates stronger robustness against distribution shifts. Direct transfer often suffers from negative transfer due to differences in time resolution (e.g. Hourly vs. Minute) or distribution drifts. Taking $ETTh1 \rightarrow ETTm2$ as an example, the DLinear model, which is sensitive to distribution shifts, performed poorly in direct transfer (MSE=0.415). LP-Mirror drastically reduced this error to 0.301, outperforming even fine-tuning directly on the target domain.

In summary, whether dealing with same-frequency transfer or complex cross-frequency scenarios (e.g. m1 to h2), LP-Mirror serves as an effective bridge, significantly improving prediction accuracy in few-shot settings by filtering for positive samples.

#### Training acceleration

Beyond improving accuracy, LP-Mirror significantly optimizes downstream training efficiency by removing harmful or redundant samples, reducing per-epoch training time, and accelerating convergence.

**Fig. 2:**
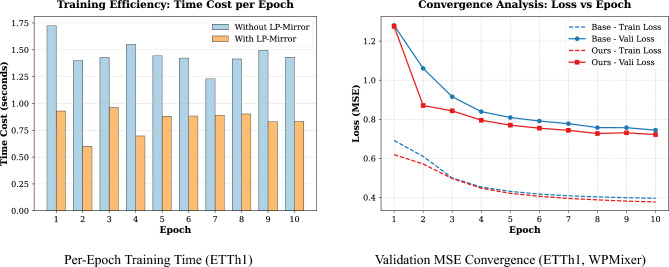
Training dynamics analysis (ETTh1). Left: After filtering with LP-Mirror, the per-epoch training time for all four backbone models decreased significantly. Right: Convergence curves show that the model converges faster to a better solution after filtering, validating the impact of data quality on training efficiency.

Regarding per-epoch training time, the reduction in data volume directly lowers computational overhead. As shown in Fig. 2a, although the data removal ratio is 30%, the actual efficiency gain exceeds 30% due to factors like data loading and warmup. For instance, at the 2nd epoch, using LP-Mirror reduced the time elapsed to nearly half that of the baseline.

Regarding convergence speed, the filtered subset of high-value data allows the model to focus on capturing core temporal patterns, reducing gradient oscillation caused by invalid samples. As illustrated in Fig. 2b, taking the WPMixer model on the ETTh1 dataset as an example, the model incorporating LP-Mirror converged significantly faster in the first two epochs compared to the baseline. Importantly, this acceleration did not compromise performance; instead, the validation MSE was further optimized.

### Superiority (RQ2)

#### Screening time overhead analysis

The core advantage of LP-Mirror lies in its extremely low computational cost, derived from the design of the lightweight linear proxy model and the mirrored influence assumption. This fundamentally avoids the expensive Hessian matrix inversion or multiple gradient retrainings required by traditional methods, achieving orders of magnitude improvement in screening efficiency. We objectively compared LP-Mirror against mainstream baselines regarding screening time overhead and downstream training acceleration across varying dataset scales.

**Fig. 3:**
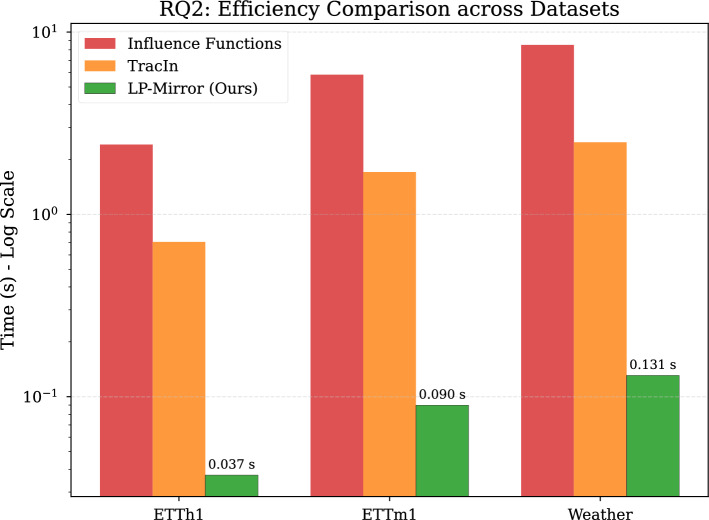
Efficiency comparison. Wall-clock time (log scale) for data screening on three datasets. Compared to Influence Functions and TracIn, LP-Mirror achieves orders of magnitude acceleration, with the advantage widening as dataset size increases.

As shown in Fig. 3, LP-Mirror’s screening efficiency significantly outperforms baselines on all datasets, with the advantage becoming more pronounced as data scale and dimensionality increase. specifically, on the small-scale ETTh1 (8,545 samples), LP-Mirror took only 0.037 seconds, far faster than other methods. With the data increasing to medium-scale ETTm1 (34,465 samples), LP-Mirror maintained a millisecond-level response (0.082 seconds). On the most challenging large-scale high-dimensional Weather dataset (36,792 samples, 21 dimensions), LP-Mirror completed screening in just 0.131 seconds, whereas TracIn took over 1 second, and Influence Functions (IF) approached 10 seconds.

#### Performance comparison

To comprehensively assess the effectiveness of LP-Mirror, we compared it against existing mainstream data selection methods. Since most existing methods are designed for Computer Vision (CV) classification tasks, we adapted their metrics and screening logic to fit time series forecasting (regression tasks).

We selected three categories of representative methods as baselines:

1. *Random Sampling:* A naive baseline that uniformly and randomly selects a subset from the training set, serving as a lower bound for performance.
2. *Loss-based Heuristics (Small-Loss):* Based on the assumption that “samples with small loss are more reliable,” this method prioritizes retaining samples with smaller MSE loss during training and removes high-loss samples (often treated as outliers).
3. *Gradient-based Classics (Influence Functions):* Selects high-influence samples by calculating the approximate impact of samples on validation set loss (using Hessian-free approximation).
4. *Recent Methods (Moderate-DS*^41^, *InfoBatch*^42^**):**
  *Moderate-DS:* Prioritizes samples with “moderate” loss, assuming they lie near decision boundaries and are most informative.
  *InfoBatch:* A dynamic pruning method that adjusts sampling probability based on loss distribution during training to balance efficiency and accuracy.

To ensure fair comparison, we unified the experimental environment for all methods. Experiments were conducted on two typical datasets, ETTm1 and Weather, representing medium and large-scale scenarios, respectively. The backbones used were WPMixer and PatchTST, with input length and prediction horizon fixed at 96. For all screening methods, we controlled the final data Keep Ratio to match LP-Mirror’s optimal ratio (70%) and evaluated them under identical hyperparameter settings (Table 5).

**Table 5:** Comparison of different data selection methods on ETTm1 and Weather datasets.

Method	WPMixer				PatchTST			
	ETTm1 (Horizon=96)		Weather (Horizon=96)		ETTm1 (Horizon=96)		Weather (Horizon=96)	
	MSE	MAE	MSE	MAE	MSE	MAE	MSE	MAE
**Full Data**	0.275	0.333	0.141	0.188	0.293	0.346	0.149	0.198
Random Sampling	0.289	0.342	0.168	0.201	0.324	0.389	0.153	0.239
Small-Loss	0.281	0.339	0.158	0.191	0.299	0.351	0.153	0.235
Influence Functions	0.267	0.334	0.152	0.193	0.290	0.341	0.151	0.184
Recent Method								
Moderate-DS^41^	0.270	0.336	0.150	0.192	0.292	0.344	0.148	0.182
InfoBatch^42^	0.265	0.333	0.149	0.191	0.289	0.339	0.147	0.180
**LP-Mirror (Ours)**	**0.261**	**0.331**	**0.149**	**0.190**	**0.286**	**0.337**	**0.146**	**0.179**

Best results are bolded.

### Mechanism analysis (RQ3)

To delve into the internal mechanism underlying LP-Mirror’s effectiveness, we conducted a comprehensive analysis from three dimensions: theoretical verification, parameter robustness, and feature visualization. The experimental design included injecting 30% Gaussian noise into training sets to simulate real anomalies, traversing different data keep ratios to test hyperparameter sensitivity, and conducting waveform visualization studies on typical samples. These analyses aim to reveal the rationality of the screening logic and its physical significance.

#### Verification of gradient consistency assumption

**Fig. 4:**
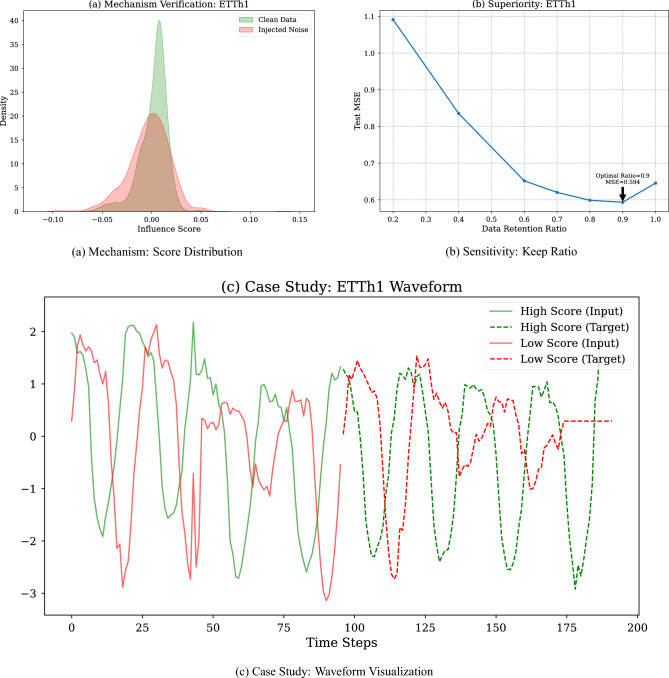
Comprehensive analysis of LP-mirror mechanism. (**a**) In the noise injection test, LP-Mirror successfully assigned negative influence scores to injected noisy samples (red area), clearly distinguishing them from clean data (green area); (**b**) Performance exhibits a “U-shaped” trend with respect to the keep ratio, peaking at 0.9; (**c**) Waveform visualization reveals that high-score samples (green) possess clear periodicity, while low-score samples (red) are mostly straight lines or abnormal fluctuations caused by sensor failure.

The theoretical cornerstone of LP-Mirror is the “Gradient Consistency” assumption. The gradient direction of beneficial samples should align with that of the guidance set. The noise injection experiment strongly supports this assumption. Figure 4a shows the sample score distribution on ETTh1 after injecting noisy samples. It is evident that the score distribution of injected noise samples shifts towards the negative direction. This result indicates that LP-Mirror’s influence score accurately quantifies the contribution of samples to the target task, ensuring the scientific validity of the screening process.

#### Parameter sensitivity and robustness analysis

The data keep ratio is a key hyperparameter in LP-Mirror. By traversing keep ratio values (from 0.1 to 1.0), we found that the test set MSE follows a clear “U-shaped” pattern (Fig. 4b). Experiments show that the optimal keep ratio stabilizes around 0.9, where the MSE reaches a minimum of 0.594, a 5.4% reduction compared to full data. When keep ratio is too low (e.g. 0.5), underfitting occurs due to the loss of massive effective samples and when keep ratio is too high (approaching 1.0), the retention of harmful samples hinders model performance.

*Robustness to guide set size* Our framework demonstrates robust empirical performance across various guide set conditions. Firstly, regarding the size of the guide set, the seven benchmark datasets in our experiments inherently employ different train/validation/test split ratios (as shown in Table ). This natural variance in the absolute and relative sizes of the validation set (which acts as the guide set in standard scenarios) does not hinder LP-Mirror from consistently delivering significant denoising improvements across all tasks. Secondly, regarding representativeness and bias, even if a diminutive guide set exhibits partial distributional bias, utilizing it to retrieve structurally consistent source samples provides crucial directional guidance. As shown in Table , this adaptively guided retrieval strategy comprehensively outperforms the “Direct Transfer” baseline (which completely lacks target guidance). This proves that utilizing a suboptimal yet directionally aligned guide set is vastly superior to blind zero-shot transfer, effectively mitigating negative transfer while resisting severe bias propagation.

#### Visualization of sample features

To intuitively explain the screening logic, we visualized typical high-score and low-score samples (Fig. 4c). High-score samples (beneficial samples) generally exhibit clear periodicity and trends, such as daily patterns in electricity load, with continuous and smooth data consistent with physical common sense. Conversely, low-score samples (harmful samples) display obvious abnormal features. This capability to distinguish based on physical characteristics proves that LP-Mirror’s screening results align highly with human intuition regarding high-quality data. It relies not only on mathematical gradient consistency but also possesses strong interpretability, providing credible assurance for practical deployment.

## Conclusion

In this work, we presented LP-Mirror, a unified and computationally efficient data selection framework tailored for time series forecasting. Addressing the pervasive challenges of data noise in large-scale datasets and data scarcity in emerging scenarios, our approach fundamentally reimagines data valuation by synergizing a lightweight Linear Proxy with the MIH.

Unlike traditional influence estimation methods that suffer from prohibitive computational costs due to Hessian matrix inversions, LP-Mirror leverages efficient forward passes to approximate sample utility. This design choice not only ensures scalability for long-sequence time series but also decouples the valuation process from the complexity of the backbone model.

The framework demonstrates a versatile dual-track capability. First, for in-domain denoising, it effectively utilizes validation guidance to identify and exorcise detrimental samples–such as outliers and mislabeled data–thereby enabling the main model to focus on high-value subsets. This results in a simultaneous improvement in convergence speed and prediction accuracy. Second, for cross-domain few-shot adaptation, the framework functions as a semantic retrieval engine. By using a minimal set of target data as a “compass”, it successfully navigates large-scale source pools to retrieve distributionally consistent samples, offering a robust solution to the cold-start problem where traditional transfer learning often fails due to negative transfer.

Extensive empirical evaluations across seven real-world datasets (covering energy, traffic, and weather) and four distinct model architectures (Linear, Transformer, and MLP-based) confirm the superiority of our method. LP-Mirror consistently outperforms both heuristic strategies (e.g. Small-Loss) and advanced selection algorithms (e.g. InfoBatch and Moderate-DS), proving its efficacy as an architecture-agnostic solution. By providing a mathematically grounded yet practically implementable tool for data optimization, this work paves the way for more reliable and efficient data-centric AI applications in time series forecasting.

## Data Availability

The datasets analyzed in this study are publicly available. We accessed the formatted versions of these datasets via the Time-Series-Library repository at https://huggingface.co/datasets/thuml/Time-Series-Library. The original datasets were introduced in their respective publications^5,12,50^. Besides, the code of our work is presented at https://github.com/LP-Mirror/LP_Mirror.
